# A Cohort Pilot Study on HIV-Associated Neuropsychological Impairments in Hemophilia Patients

**DOI:** 10.3389/fnhum.2015.00313

**Published:** 2015-06-02

**Authors:** Silvia Riva, Ilaria Cutica, Caspar Krampe, Laura F. Reinecke, William Russell-Edu, Cristina Santoro, Angiola Rocino, Elena Santagostino, Vega Rusconi, Gabriella Pravettoni

**Affiliations:** ^1^Department of Health Sciences, University of Milan, Milan, Italy; ^2^European Institute of Oncology (IEO), Milan, Italy; ^3^Haemophilia and Thrombosis Centre, Umberto I Hospital, Rome, Italy; ^4^Haemophilia and Thrombosis Centre, S. Giovanni Bosco Hospital, Naples, Italy; ^5^ABB Haemophilia and Thrombosis Centre, IRCCS Ca’Granda, Milan, Italy

**Keywords:** hemophilia, HIV, neuropsychological impairments, cognitive disorders, neuropsychological assessment

## Abstract

Despite advances in the management of HIV infection with the introduction of combination antiretroviral therapy, it is well known that HIV can directly infect the central nervous system and, as a result of such infection, neuropsychological impairments can be manifested. In this study, we tried to determine whether seropositivity was associated with a poor neuropsychological performance in patients with hemophilia and HIV. Such a cohort of patients is very often underrepresented and understudied in the HIV literature. To amend such a gap, we carried out an extensive neuropsychological evaluation on these patients, and compared their performance with that of a group of seronegative hemophilia patients. The results revealed that HIV infection in HIV-seropositive (HIV+) hemophilia patients was associated with deficits in attention, short-term memory, abstraction, and visual recognition. Such results are still preliminary and explorative due to the small cohort of patients enrolled. However, the results do seem to have some important implications for day-to-day functioning, as the level of impairment detected may cause difficulties in completing common everyday tasks such as maintaining adherence to complex medication regimens or maintaining social life activities. Continued research into the mechanisms related to HIV and neurocognitive dysfunction may provide targets for interventions that could have meaningful consequences in the real world for HIV hemophilia patients.

## Introduction

Although severe forms of HIV-associated neuropsychological impairments have decreased since the widespread use of combination antiretroviral therapy (cART), such impairments continue to persevere among patients with HIV infection (Heaton et al., 2010; Becker et al., 2011; Grant et al., 2014). Different elements contribute to the development and severity of neuropsychological impairments, such as potentially irreversible brain injury that occurred before patients were started on cART, as well as imperfect blood–brain barrier penetrance leading to inadequate suppression of the adverse effects of HIV on central nervous system (CNS) function [for review, see Heaton et al. (2011)]. In addition, there is a high prevalence of comorbid conditions and confounding factors that may interfere with the effects of HIV on the CNS (Weiss et al., 2010), such as CD4 nadir count (Munoz-Moreno et al., 2008; Ellis et al., 2011; Heaton et al., 2011), the time of infection (Ettenhofer et al., 2009), the presence of other infections such as hepatitis C virus, and the use of drugs (Clifford et al., 2005; Shimizu et al., 2011).

Current estimates indicate that as many as 50% of HIV+ individuals display some degree of neuropsychological impairment (Dawes et al., 2008; Giesbrecht et al., 2014) when impairment is derived from comparisons with normative performance standards (e.g., Heaton et al., 2011; Giesbrecht et al., 2014). Nevertheless, a recent meta-analysis revealed lesser attentional, motor, and executive skill impairments in HIV+ individuals treated with cART (Al-Khindi et al., 2011). Although neuropsychological profiles vary among HIV+ individuals (Giesbrecht et al., 2014), deficits in speed of information processing (Llorente et al., 1998; Carey et al., 2004, 2006; Giesbrecht et al., 2014), fine motor speed and dexterity (Chang et al., 2002; Judd et al., 2005), aspects of learning and memory (Peavy et al., 1994; Carey et al., 2006; Martin et al., 2007; Maki et al., 2009), especially verbal memory (Seider et al., 2014) and prospective memory (Doyle et al., 2013), abstraction (Heaton et al., 1995), and attention (Heaton et al., 1995; Hardy and Hinkin, 2002; Levine et al., 2008). Multiple domains of executive functioning, such as cognitive flexibility, decision-making, and planning (Chang et al., 2002; Iudicello et al., 2008; Munoz-Moreno et al., 2008; Cattie et al., 2012), have also been commonly identified and described.

Despite significant advances in our knowledge over the past 20 years regarding the prevalence, clinical correlates, and functional impact of HIV-associated neuropsychological impairments, we still know very little about the different types of neuropsychological profiles, which are HIV-associated in different populations. A limitation of the existing literature examining cognitive disorders in HIV is the underrepresentation of certain types of infected populations with HIV. Neuropsychological impairments have been mainly studied in HIV-seropositive (HIV+) patients infected vertically or postnatally during the earliest period of life of the child (e.g., through breast milk) or in specific cohorts of adults such as drug users. Both such macro-categories present confounding factors that may also affect cognitive performance, therefore making it difficult to identify the cognitive impairments attributable to HIV infection. Indeed, it has been shown that children infected vertically or immediately postnatally present adjunctive disorders (Isanaka et al., 2009; Siegfried et al., 2011), such as HIV-related dysfunctions in energy balance (Henderson et al., 1994; Mulligan et al., 1997; Johann-Liang et al., 2000; Batterham, 2005), neuro-developmental growth changes (Rondanelli et al., 2002; Van Rossum et al., 2003), and neurological changes (Reger et al., 2002; Heaton et al., 2004; Antinori et al., 2007). In the cohort of drug users, patients show different neuropsychological impairments; however, these profiles are often difficult to describe because they are complicated by a myriad of confounding factors such as the abuse of substances as alcohol, cocaine, and opiates (Starace et al., 1998; Lundqvist, 2010; Buttner, 2011), emotional disorders, such as anxiety and depression (Byrd et al., 2013), and different risk behaviors (De Ronchi et al., 2002).

While cognitive disorders in HIV are prolific in these areas of research, they remain comparatively understudied in other cohorts of patients, for example, patients who were medically induced with HIV infection through blood transfusions. Such patients represent an interesting cohort because they were generally infected postnatally at an older age, and because they generally do not present the confounding factors of drug-user populations (Rubin et al., 1999; Brown et al., 2000; Ettenhofer et al., 2009). Among these patients, those with hemophilia represent a particularly interesting cohort of patients (Riva et al., 2014a).

### Hemophilia

Hemophilia is a rare coagulation disorder in which a crucial clotting factor in blood is missing either partly or completely. Being a sex chromosome-gene-related bleeding disorder, it occurs primarily in the male population (White et al., 2001). The clinical hallmark of hemophilia is recurrent spontaneous bleeding, most frequently in joints such as the ankles, elbows, and knees, as well as in muscles (Kern et al., 2004; Muça-Perja et al., 2012; Gringeri et al., 2013). The treatment of hemophilia is based on the replacement of the missing clotting factor when bleeding occurs (on-demand treatment) or on a regular and continuous basis (prophylactic treatment) (Santagostino and Mannucci, 2000). In the western world, prophylactic treatment of young hemophiliac patients is considered as current best practice (Krasuska et al., 2012; Santoro et al., 2013; Franchini et al., 2014). Generally, prophylaxis with clotting factors is initiated in childhood after the first joint bleed by the age of 2–4 years (Santagostino and Mannucci, 2000; Makris, 2012; Richards et al., 2012). One of the greatest risks faced by those with hemophilia in the late 1970s and early 1980s was the possibility of contracting HIV, commonly known as the AIDS virus (Franchini and Mannucci, 2012; Mangiafico et al., 2012). This happened when people received transfusions of clotting factors drawn from infected blood. It is estimated that 60–70% of people with hemophilia were infected with HIV in this way between 1979 and 1985 (Mannucci, 2005). In those years, blood donations were not tested for HIV and people with hemophilia were especially likely to get infected for two reasons: they received many transfusions, and each transfusion contained pooled clotting factors drawn from the blood of numerous donors in order to obtain enough clotting factors to be effective. If any one of those donors happened to be infected with HIV, then the hemophiliac was at risk.

For this reason, the cohort of patients who have both hemophilia and HIV represents a different and interesting cohort of HIV patients to be evaluated in relation to neuropsychological impairments.

However, studies on HIV+ hemophiliacs and neuropsychological impairments are sparse and not very recent. The most important investigation in this field was the Hemophilia Growth and Development Study (HGDS) by Hilgartner et al. (1993). The HGDS was a multicenter study of the long-term effects of HIV infection on growth and neurodevelopment in HIV+ hemophiliac children and adolescents most of whom were asymptomatic for HIV disease at baseline, in comparison to a group of HIV− hemophiliacs of similar age. Longitudinal and prospective data from the HGDS identified some specific impairments at the memory level (Loveland et al., 2000; Nichols et al., 2000), attention (Watkins et al., 2000), and language. These impairments, however, were not clearly described in relation to HIV infection but as neuropsychological impairments related to socio-educational covariates such as school absenteeism and poorer academic achievement, which frequently marked these young patients.

The other few studies have identified cognitive impairment in HIV+ hemophiliacs as related to the decrease in immunological functioning, especially when the CD4+ cell count is lower than 200. For example, Riedel et al. (1992) identified a linear association between neuropsychological impairment in attention, motor skills, visual performance, and the decrease of CD4+. Similarly, Blanchette et al. (2002) identified impairments in motor speed and fine motor skills, and Nichols et al. (2000) concluded that HIV+ hemophiliacs presented deficits in communication skills in relation to their immune-functioning system.

Although there are some studies available in the context of a pediatric population, studies are totally underrepresented in the adult population with mixed and confusing results (Turnbull et al., 1991; Riedel et al., 1992). The only two exceptions are the work of Siboni et al. (2009) and Zanon et al. (2014). The first work compared the Mini-Mental State Examination (MMSE) in a hemophiliac HIV− population with the general population, in which differences were not found to be significant. Similarly, the second work determined that HIV− adult hemophiliacs presented a normal overall cognitive performance as measured by the MMSE >25 (Folstein et al., 1975). Alterations occurred only in some patients and they were associated with the presence of spontaneous brain hemorrhage (microbleeds).

However, no prospective studies about adults HIV+ hemophiliacs are currently available.

Therefore, understanding the temporal course and the mechanisms of cognitive impairment in this cohort of patients represents an important goal of current research in order to broaden and deepen our current understanding of the impact of HIV on postnatal growth.

Considering the lack of research consensus about the type of neuropsychological impairments and the scarcity of research in the context of hemophilia, the present study aimed to assess the presence and the extent of neuropsychological impairments in a randomly selected cohort of HIV+ adult hemophiliacs comparing the performance of this cohort with that of a control group composed of HIV− hemophiliacs.

## Materials and Methods

### Participants

Fifteen HIV+ hemophiliacs (experimental group) and 30 HIV− hemophiliacs (control group) were administered neurocognitive batteries between December 2012 and December 2013. Consecutive patients were recruited through their treating physicians via the hemophilia and thrombosis outpatient clinics for three Italian centers. All hemophiliacs had adequate Italian language fluency for the purpose of valid psychometric testing. The inclusion criteria for the experimental group were as follows: diagnosis of hemophilia, diagnosis of HIV with CD4+ counts consistently >200 cells/mm^3^, treatment with cART, age >18 years. Exclusion criteria included diagnosis of AIDS, serious mental illness with a certificated diagnosis (e.g., major depression, anxiety, bipolar disorder), or known CNS pathology, including progressive multifocal leukoencephalopathy, cancer affecting the brain, neurosyphilis, active cytomegalovirus infection, multiple sclerosis, stroke, and seizures/epilepsy. Control patients had a diagnosis of hemophilia and were tested for HIV antibody upon recruitment to the study unless they had a negative test in the past 3 months with no other illness during this time period.

Neurocognitive assessment was conducted by the first author (Silvia Riva) in Milan and Naples and by Silvia Riva and Ilaria Cutica (co-authors) in Rome. Table 1 summarizes the patients enrolled in the three hospitals.

**Table 1:** **Participants characteristics**.

	HIV-group (*n* = 30)		HIV+ group (*n* = 15)		Sig.
	*n*	%	*n*	%	
Highest educational attainment					0.060 ^a^
Primary school	3	10	1	7	
Secondary school	3	10	8	53	
High school	18	60	5	33	
University	6	20	1	7	
Currently in professional occupation					0.255 ^b^
Yes	23	77	9	54	
No (unemployed/disability pension)	7	23	6	46

	**M**	**SD**	**M**	**SD**	

Age	44	8.9	45	8.4	0.446 ^a^

*^a^Mann–Whitney *U* test*.

*^b^Chi-square test*.

### Ethics statement

Written informed consent was obtained from each patient. The study was approved by the institutional ethical committees of the three Italian centers involved in this study: the IRCCS Ca’Granda (Milan), The Umberto I Hospital (Milan), and the S. Giovanni Bosco Hospital (Naples) (reference number of IRCCs Ca’Granda, coordinator center: 18092012). The study was conducted in accordance with the Helsinki Declaration (59th WMA General Assembly, Seoul, 2008).

### Neuropsychological assessment

Patients completed an extensive neuropsychological test battery measuring five major cognitive domains. *Attention* (1) was assessed by the Trail Making Test (TMT), which is a neuropsychological test of visual attention and task switching (Tombaugh Nichols et al., 2000). *Memory* (2) was assessed by the Letter–Number Sequencing of the Weschler Adult Intelligence Scale, third edition (WAIS-III) (Wechsler, 1995), which measures the number storage capacity of the working memory (Digit span memory). *Language Comprehension* (3) was assessed with the short version of the Token Test, which is a valuable measure for assessing receptive language (Spellacy and Spreen, 1969). *Visual recognition* (4) was assessed by the Rey Tangled Lines Task (Rey, 1964), a visual tracking and visuomotor processing task. Finally, *executive functions* (5) were assessed widely by the Clock-drawing Test (Freedman et al., 1994), which is a widespread test used for screening cognitive impairments and dementia and measures spatial dysfunctions and neglect, by the Verbal Fluency test (Lezak, 1995) designed to test phonemic memory in which participants have to say as many words as possible from a category in a given time (60 s), and by the Italian Test of abstraction of the Brief Neuropsychological Exam (Mondini et al., 2003) aimed at detecting the ability of logical reasoning and generalization of concepts.

### Procedure

After pre-selection of patients, patients were asked to give their written consent to data collection by signing the data protection form. Neuropsychological profiles were based on neuropsychological assessment and recorded by means of documentation forms that were completed by the research psychologist (Silvia Riva). Patient status (HIV+ or HIV−) was blinded to the examiner. The examiner was not blinded only for the data analysis phase.

Overall patient clinical status was evaluated at routine medical appointments, including presence of concomitant diseases and the monitoring of safety variables (e.g., adverse events).

### Sample size determination

The optimal sample size was calculated through analyzing and integrating related publications on cross-sectional studies (Riedel et al., 1992; Blanchette et al., 2002) via effect sizes (*F*-values respective η-square-values, Cohen’s *d*, and Λ for multivariate approaches) with G*Power (Faul et al., 2007). A synopsis of needed sample sizes for the different articles and different outcome measures within the articles was constructed and evaluated. This resulted in an approximate value of the minimum total sample size of 10 cases and 20 controls with a case-control ratio of 1:2. There were two essential reasons for considering the 1:2 matching scheme: (1) concern for sufficient numbers in stratified analysis; (2) increasing power given the expected prevalence (Hennessy et al., 1999). Overall, we enrolled 45 patients: 15 cases and 30 controls.

The sample size calculation took into account the prevalence of hemophilia as a rare disease (10 per 100,000 males born) and the incidence of HIV (currently, around 70% of diagnosed hemophiliac patients are HIV+ because of receiving infected blood products prior to the availability of HIV screening) (Clinical and Epidemiological Key Elsevier, 2014).

In order to avoid bias in the inclusion phase, we collected a complete list of the patients, and chose patients via the use of a random number generator. HIV− hemophiliacs were matched with HIV patients by age (±5 years).

### Statistical analysis

All comparisons made between HIV+ and HIV− groups were conducted on raw cognitive data. For conventional neurocognitive measures (Trail Making Test, Digit Span, Verbal Fluency Test), performance of the HIV+ group was further inspected in the context of published normative data using the following procedures. First, each HIV+ participant’s raw score was converted to its corresponding age-corrected standard score based upon the published norm-referenced data.

Given the relatively small number of participants, the non-normality of several distributions and the unbalanced groups, we decided to use the exact methods with Monte–Carlo approximation: a series of non-parametric statistical algorithms that enable researchers to make reliable inferences when data are sparse, heavily tied or unbalanced, not normally distributed, or fail to meet any of the underlying assumptions necessary for reliable results using the standard asymptotic method (Siegel and Castellan, 1988). The exact methods with Monte–Carlo approximation used for comparisons were the Wilcoxon–Mann–Whitney Test for continuous measures, and Chi-square for categorical variables (α = 0.05, two-tailed).

Finally, one-way ANCOVAs were conducted to compare the scores between the two groups on raw data of neuropsychological measures that were obtained in both groups, co-varying for age, education, and working status.

## Results

### Patients characteristics

HIV+ and HIV− hemophiliac patients were matched on age; however, no significant differences were found for other socio-demographic variables, such as education, and working status. It is worth noting the frequency of patients with disability pension or unemployed (higher percentage in the HIV+ group) and the smaller presence of patients with a high level of schooling (particularly in the HIV+ group). Details are presented in Table 1.

### Neuropsychological assessment results

Scores on the cognitive domains and on each individual test (raw test scores ± SD for tests) of the two groups are shown in Table 2. Subsequent analyses of the individual domains showed a significantly worse performance of HIV+ hemophiliacs on the domains *Attention* (Trail Making A test, *U* = 104.50, *z* = −2.80, *p* = 0.005; Trail Making B test, *U* = 107.00, *z* = −2,73, *p* = 0.006), *Memory* (Digit Span memory, *U* = 141.00, *z* = −2.06, *p* = 0.039), and *Abstraction* (*U* = 144.00, *z* = −2.06, *p* = 0.039), and *Visual recognition* (*U* = 115,00, *z* = −2.56, *p* = 0.010).

**Table 2:** **Neuropsychological test results on the different cognitive domains and on each test for the HIV− and the HIV+ hemophiliac groups**.

Cognitive domains	HIV-group (*n* = 30)	HIV+ group (*n* = 15)	*p*-Value	*r* (*Z*/√*N*)^*^
	Mean (SD)	Mean (SD)	
**Attention**
Trail Making Test A	40.38 (±12.91)	60.93 (±24.52)	0.005	0.45
Trail Making Test B	128.66 (±57.38)	173.53 (±63.39)	0.002	0.47
**Memory**
Digit span memory	4.90 (±1.34)	3.93 (±1.71)	0.039	0.34
Phonemic memory	31.45 (±6.13)	27.53 (±6.20)	0.619	0.01
**Abstraction**
Test of abstraction	5.13 (±1.19)	4.40 (±1.29)	0.39	0.34
**Language**
Token Test	4.97 (±0.18)	4.73 (±0.79)	0.131	0.03
**Visual recognition**
Rey Tangled Lines Task	3.54 (±0.83)	2.67 (±1.17)	0.008	0.39
**Executive function**
Clock-drawing Test	7.27 (±1.80)	6.77 (±1.20)	0.340	0.02

*Test scores are presented as mean raw test scores ± SD*.

*Mann–Whitney *U* test with Monte–Carlo *p* estimation between groups*.

*r = effect size. Small size = 0.1; medium size = 0.3; large size = 0.5.

### HIV+ patients’ neuropsychological impairments relative to normative-referenced standards

When the HIV+ hemophilia patients’ neuropsychological performances were indexed in accordance with published normative-referenced standards, the magnitude of impairments appeared comparable to data estimated on raw scores in relation to *Executive functions* (Phonemic memory) (*U* = 217.00, *z* = −0.013, *p* = 0.990) and to *Attention* (Trail Making A: *U* = 110.00, *z* = −2.79, *p* = 0.005; Trail Making B: *U* = 150.00, *z* = −2.10, *p* = 0.005).

The only exception was for the task of *Memory*, where the magnitude of impairment was not significant (*U* = 183.00, *z* = −1.68, *p* = 0.286).

### ANCOVA and interactions

In addition to analyzing overall neuropsychological impairment, we were interested to determine whether any specific areas of neuropsychological performance were impacted by the interaction of age, educational level, and working status. This was accomplished by ANCOVA, controlling for the effect of age, education, and working status.

The analysis controlled for age and education and working status revealed that the HIV+ hemophiliac group performed worse than the HIV− hemophiliac group on *Attention* with regard to the Trail Making A Test (*F* = 12.379, *p* = 0.001), on *Executive Functions* with the test of Abstraction (*F* = 5.274, *p* = 0.027) and *Visual recognition* (*F* = 5.583, *p* = 0.022).

However, the predicted main effect of age was not significant (*F* = 2.00, *p* = 0.16, $\eta_{\text{p}}^{2}=0.003$), neither was the predicted main effect of education (*F* = 3.25, *p* = 0.072, $\eta_{\text{p}}^{2}=0.004$), and nor was the predicted main effect of working status (*F* = 3.33, *p* = 0.081, $\eta_{\text{p}}^{2}=0.008$). The interaction among age, education, and working status was also not significant (*F* = 0.016, *p* = 0.90, $\eta_{\text{p}}^{2}=0.001$).

Although the introduction of covariates reduced the distance between the two groups of hemophiliacs (HIV+ and HIV−), they did not eliminate the effect of HIV infection on neuropsychological impairment completely. Therefore, the presence of these covariates (age, educational level, and working status), even though they did have an impact, did not explain group differences *per se*.

## Discussion

Although neuropsychological impairments are well-described in some specific HIV populations, very few studies have investigated the cohort of patients with hemophilia and HIV. In addition, they have often yielded inconsistent results, mainly due to methodological differences. Furthermore, only a few such studies were performed in the cART era.

Moreover, as most of them were conducted on pediatric populations and not on adults, the generalizability of their findings is highly impaired. All these factors limit the generalizability of findings across this relatively small body of work.

By contrast, our current pilot study carefully defined and investigated cognitive disorders using standardized clinical tasks in a defined, well-characterized cohort of patients with HIV. This pilot examination revealed that in a small but well-controlled sample, it was possible to detect some signals of neuropsychological impairment in the context of hemophilia patients with HIV. More specifically, the results of this study showed a worse performance on neuropsychological assessment in our sample of HIV+ hemophiliacs compared with the control group of HIV− hemophiliacs, when combining all tests in one overall analysis.

The HIV+ hemophiliacs had significantly lower scores on tests of attention, memory, executive function (abstraction test), and visual recognition than did the control group. However, when adjusted for age, education, and working status, the real distance between the two groups reduced, and the difference on memory revealed to be not significant. The observation of an HIV effect on tests requiring rapid processing of information (attention) is consistent with the classic conceptualization of HIV as a subcortical disease targeting frontal–striatal circuits supporting these abilities (Reger et al., 2002; Baldewicz et al., 2004).

The finding of a decline in abstraction function is unique in the cohort of HIV+ hemophilia patients even though it has been observed in the general HIV population (Heaton et al., 1995).

Impairments in language were not found in our study. The absence of language disorders appears to contrast slightly with the main research on cognitive disorders in hemophilia, namely that conducted in the HGDS. Indeed, in the HGDS, the authors found the presence of language disorder in HIV+ pediatric patients but concluded that this disorder was associated with socio-educational factors, since these patients were more exposed to school absenteeism and lower school participation due to the management of their physical conditions requiring frequent medical examinations. However, our results cannot be completely compared with those of the HGDS as our sample was composed of adult patients and not of children or adolescents.

Regarding the ANCOVA analysis, we found a correlation between cognitive disorders and socio-demographic factors (age, education, and working status) as reported in the HIV literature. Even this result is innovative in the field of hemophilia and HIV in the adult population, especially regarding working status. However, these differences cannot be totally attributable to these covariates and it is reasonable to attribute a weight to the HIV infection.

Future comprehensive studies, however, are expected to explore the above-mentioned relationship and to clarify more concisely the weight of each confounding factor, not only cross-sectionally but also longitudinally.

Taken together, these findings, while preliminary, highlight important considerations for the design of clinical research studies exploring the cognitive effects of HIV in hemophiliacs. The current study adds to the growing literature on HIV+ hemophiliacs and cognition in the cART era and was strengthened by selective inclusion criteria (such as the level of CD4 >200, the absence of a psychiatric disorder, such as a diagnosis of depression/anxiety), thereby allowing the evaluation of a precise and representative cohort. Previous studies either lacked a matched control group or examined cognition in isolation. One of the main strengths of the current study is that it is one of the few to examine cognitive impairments in the cohort of hemophilia patients in adulthood and provides an update to reference studies, which are not very recent in this field. Also, studies in European samples of HIV patients are very scarce. This study represents one of the firsts European cohort study in hemophilia with HIV patients exploring the level of cognitive impairments.

Another strength is that this study provides an overview of the social situation of these patients who live with another chronic condition, hardly ever reported in the literature. Patients with HIV+ hemophilia present a higher rate of unemployment status, and a lower level of schooling, which probably leads to more difficulties in finding a job (Chernoff et al., 2010). Rates of unemployment in HIV+ hemophiliacs in this study were comparable to those in chronic illnesses with a recognized disability such as rheumatoid arthritis (Wallenius et al., 2009) but lower than those in other disabling conditions such as multiple sclerosis (Khan et al., 2009). This scenario must also be considered seriously from a social and health policy perspective. A multidisciplinary approach is likely to be needed, addressing both physical and psychological barriers to working, including overcoming perceived barriers to work, increasing confidence and motivation to work, and facilitating skills training and work placement (Kielhofner et al., 2004). The potential for the doctor–patient relationship (Riva et al., 2012; Riva et al., 2014b) in HIV to help promote a return to employment should be considered, with discussion forming a part of the routine HIV consultation.

As with all research, several limitations should be noted. The current study was not designed to longitudinally track the effects HIV on cognitive functioning. Future longitudinal studies may investigate more, in-depth, the presence of cognitive impairment in this population and the relationship with antiretroviral medications in order to evaluate whether and how the value of long-term treatment versus short-term treatment impacts differently on cognitive impairment. Moreover, this study was not additionally designed to compare HIV− hemophiliacs with a parallel sample of the Italian general population because, as shown by the existing literature, the cohort of HIV− hemophiliacs seems not to show any evident cognitive decline compared with the normal population (Siboni et al., 2009). However, a comparison with a specific sub-group from the general population would have further enhanced the study.

This study is limited by its design as a pilot and suffers from methodological challenges, such as the potential instability of the data related to the small sample size. However, the presence of statistically significant results renders the findings even more provocative. Future studies, with larger samples, should be completed to replicate these findings and to test for potential interaction effects among variables of interest. Another limitation is the lack of data on MRI abnormalities and other neurological indicators, which made it impossible to link the neuropsychological assessment results with some neurobiological correlates or other medical observations.

In conclusion, this cross-sectional pilot study showed decrements in the neuropsychological profile, specifically in attention, memory, executive function (abstraction test), and visual recognition in a small, but representative, randomly selected sample of hemophilia patients with HIV+ in comparison with the control group of HIV− hemophilia patients. The findings of the current study indicate that future research should focus on further unraveling the underlying mechanism of neuropsychological impairments, using neuropsychological tests, in combination with neuroimaging techniques in a larger sample.

Finally, the neuropsychological impairments detected in this study have important implications for day-to-day functioning. The level of impairment detected may cause some problems in completing common everyday tasks such as maintaining adherence to complex medication regimens (both for hemophilia and HIV), as well as holding down a full-time job. These impairments, in combination with hemophilia, may make some activities of self-care more cumbersome. As recently pointed out by the work of Fazeli et al. (2014) on HIV patients with associated neurocognitive disorders, working and active engagement in life tend to strengthen neurocognitive functioning, “by enhancing cognitive and/or brain reserve” (p. 234). Continued research into the mechanisms related to HIV and neurocognitive dysfunction may provide targets for interventions that could have meaningful consequences in the real world.

## Author Contributions

RS, as first author, developed the design of the study, carried out the study, performed the statistical analysis, and wrote the manuscript. IC participated in carrying out the study and drafted the manuscript. CK and LR participated in statistical analyses and drafted the manuscript. RESW revised linguistically our paper. CS participated in carrying out the study in Rome. AR participated in carrying out the study in Naples. VR participated in carrying out the study in Milan. GP helped the first author in developing the study and participated in writing of the paper. All authors read and approved the final manuscript.

## Conflict of Interest Statement

The authors declare that the research was conducted in the absence of any commercial or financial relationships that could be construed as a potential conflict of interest.
